# “Surviving is not enough”: shifting the focus from treatment success to quality of life in anal cancer survivors. Patient- reported outcomes and the evolving landscape of survivorship care

**DOI:** 10.3389/fonc.2026.1761654

**Published:** 2026-05-19

**Authors:** Stefania Manfrida, Natalia Barogi, Viola De Luca, Diana Giannarelli, Loredana Dinapoli, Bruno Fionda, Giuditta Chiloiro, Fabio Marazzi, Daniela Pia Rosaria Chieffo, Luca Tagliaferri, Maria Antonietta Gambacorta

**Affiliations:** 1 Department of Radiology and Radiation Oncology, Fondazione Policlinico Universitario Agostino Gemelli Istituto di Ricovero e Cura a carattere Scientifico (IRCCS), Roma, Italy; 2 Epidemiology and Biostatistics Facility, G-STeP Generator, Fondazione Policlinico Universitario Gemelli Istituto di Ricovero e Cura a carattere Scientifico (IRCCS), Rome, Italy; 3 Clinical Psychology Unit, Fondazione Policlinico Universitario Gemelli Istituto di Ricovero e Cura a carattere Scientifico (IRCCS), Rome, Italy; 4 Department of Women, Children and Public Health, Università Cattolica del Sacro Cuore, Rome, Italy; 5 Università Cattolica del Sacro Cuore, Rome, Italy

**Keywords:** anal canal cancer, long-term effects, patients’ reported outcomes (PROMs), quality of life, radiation therapy

## Abstract

**Background:**

Chemoradiotherapy (CRT) is the standard treatment for squamous cell carcinoma of the anal canal (ACC), achieving excellent local control and sphincter preservation. However, many long-term survivors experience persistent bowel, urinary, sexual, and psycho social sequelae affecting quality of life (QoL). The PROACT study (Patient – Reported Outcomes in Anal Cancer Patients Treated with Intensity-Modulated Radiotherapy; NCT06364579) wants to explore the relationship between oncologic outcomes and patient-reported QoL in the era of modern radiotherapy.

**Methods:**

This single-institution ambispective study included patients with a diagnosis of ACC treated between 2011 and 2024 with intensity modulated radiotherapy (IMRT)-based CRT, followed—when indicated—by an image guided interventional radiotherapy (IRT) boost. Oncologic outcomes and toxicity were assessed using standard criteria. QoL was evaluated annually up to 5 years post-treatment using the EORTC QLQ-C30 and anal cancer–specific QLQ-ANL27 questionnaires. Statistical analyses explored associations between QoL domains and patient-, disease-, and treatment-related factors.

**Results:**

Median age was 62 years (range 34–83); 82.2% were female. Median follow-up was 51 months. Three- and five-year overall survival were both 97.5%. Disease-free survival was 88.2% and 84.5% at three and five years, respectively, while locoregional relapse-free survival was 92.8% and 89.0%. Colostomy-free survival showed identical rates at three and five years (97.3%). Late≥ G3 Gastrointestinal toxicity occurred in 6 patients (6.7%). Compared with the general population, both sexes reported significantly higher global QoL (males *p* = 0.002; females *p* = 0.001), while diarrhea was worst in women (*p* = 0.0008). Younger age (<70 years), female sex, and late GI toxicity correlated with poorer functional outcomes, particularly in bowel and sexual domains.

**Conclusions:**

PROACT underscores that treatment success in anal cancer extends beyond cure, encompassing survivorship, functionality, and well-being. Integrating oncologic and patient-reported outcomes offers a comprehensive, patient-centered framework for optimizing long-term care.

## 1 Introduction

Squamous cell carcinoma of the anal canal (ACC) is predominantly managed with definitive chemoradiotherapy (CRT), which provides excellent local control and allows sphincter preservation in the majority of patients (1). While oncologic outcomes have steadily improved, many long-term survivors experience persistent bowel, urinary, sexual, and psychosocial dysfunction, resulting in a substantial and often underrecognized burden on quality of life (QoL) (2–4).

In this context, ACC can be considered a function-sensitive disease, in which treatment-related sequelae directly impact daily activities, social functioning, and overall well-being. Modern radiotherapy innovations—particularly intensity-modulated radiotherapy (IMRT) and volumetric modulated arc therapy (VMAT)—have enhanced dose conformality and reduced treatment-related toxicity compared with historical techniques (5, 6).

However, the extent to which these advancements translate into better long-term functional outcomes remains unclear. To date, most available evidence relies on clinician-reported toxicity, typically assessed using the Common Terminology Criteria for Adverse Events (CTCAE). While essential for standardized reporting, CTCAE-based evaluations reflect the presence and severity of symptoms from a clinician perspective and may underestimate the subjective burden experienced by patients in everyday life.

In contemporary IMRT-based series, late toxicity is generally reported as relatively limited, with approximately 55% of patients experiencing ≤ Grade 2 events and only around 16% Grade 3 toxicity (5).

However, these reassuring toxicity profiles contrast with patient-reported outcomes, as prior evidence has demonstrated only fair-to-moderate agreement between clinician-reported toxicity and patient-reported experience, with relevant functional impairments often underrecognized (28). Notably, Lefevre et al. further showed that multiple functional domains significantly worsen at 1 year after treatment, including bowel, sexual, and urinary function. In particular, symptoms such as unintentional flatulence and fecal incontinence (bowel), dyspareunia and erectile dysfunction (sexual), and dysuria and urinary incontinence (urinary) were significantly impaired (p <.001), highlighting a clinically relevant mismatch between clinician-reported toxicity and real-life functional outcomes.

These findings underscore the need for systematic integration of patient-reported outcome measures (PROMs) into anal cancer research, particularly in the context of modern radiotherapy, where traditional toxicity metrics alone may fail to capture the true burden of survivorship.

Patient-reported outcome measures (PROMs) are therefore crucial to capture patients’ own perspectives regarding the physical, emotional, and social effects of treatment, yet their use in anal cancer research remains limited (7). A recent systematic review by Sterner et al. (8) underscored substantial heterogeneity in QoL studies for anal cancer and highlighted the absence of disease-specific, standardized PROM-based assessments.

Importantly, while generic QoL instruments (e.g., EORTC QLQ-C30) are valuable for assessing global health status and broad functional domains, they may not fully capture anal cancer–specific issues. Disease-specific PROMs, such as the EORTC QLQ-ANL27, are therefore essential to adequately assess specific key domains.

These limitations restrict the ability to fully define survivorship in the modern radiotherapy era and to identify modifiable factors influencing long-term outcomes.

The PROACT study (Patient-Reported Outcomes in Anal Cancer Patients Treated with IMRT; NCT06364579) was designed to address this critical need by prospectively evaluating clinical outcomes and QoL in patients treated with modern IMRT-based CRT. By integrating validated PROMs with oncologic endpoints. PROACT seeks to develop a more comprehensive understanding of survivorship and to inform patient-centered care strategies that balance therapeutic efficacy with preservation of long-term health-related quality of life. Ultimately, redefining treatment success for ACC requires shifting focus beyond tumor control to encompass sustained physical function, emotional resilience, and social participation in daily life.

## 2 Materials and methods

### 2.1 Study design

This ambispective single-institution observational study included 90 consecutive patients treated between 2011 and 2024 with IMRT-based chemoradiotherapy with histologically confirmed, non-metastatic squamous cell carcinoma of the anal canal.

All patients provided written informed consent. Patient-reported outcomes (PROMs) were collected prospectively starting from January 2023.

For newly diagnosed patients, questionnaires were administered from baseline (at or before treatment initiation) and during follow-up according to the study schedule.

In addition, patients already in follow-up at the time of study activation were included and completed the questionnaires during follow up visits, providing cross-sectional data on long-term survivorship.

This combined approach allows the evaluation of both longitudinal QoL trajectories in newly treated patients and long-term outcomes in previously treated patients.

Baseline staging included pelvic MRI with contrast, contrast-enhanced CT of the chest, abdomen, and pelvis, and ^18F-FDG PET-CT, according to AJCC/UICC 8th edition (2017) criteria.

### 2.2 Patient population

The study included patients with histologically confirmed squamous cell carcinoma of the anal canal treated with curative-intent chemoradiotherapy using IMRT with at least a 1 year follow-up. The 1-year cutoff was selected to exclude the early post-treatment phase and to focus on patients beyond acute and subacute toxicity.

Inclusion criteria were: age ≥18 years, ECOG performance status 0–3, and indication for curative-intent chemoradiotherapy.

Exclusion criteria included: age <18 years, ECOG performance status >3, and metastatic disease at diagnosis not amenable to curative-intent treatment.

### 2.3 External beam radiotherapy, concomitant chemotherapy and Interventional radiotherapy (IRT- brachitherapy)

All pts underwent CRT. Concomitant chemotherapy consisted of 5-fluorouracil (1000 mg/m² on days 1–4 and 29–32) and mitomycin C (10 mg/m² on days 1 and 29), following the FUMIR schedule. Radiotherapy was delivered with IMRT according to institutional protocols (9), applying standard criteria for patient setup, target delineation, and dose prescription.

Target volume delineation was based on clinical examination and imaging (pelvic MRI and/or PET/CT when available). The gross tumor volume (GTV) included the primary tumor and involved lymph nodes.

The clinical target volume (CTV) encompassed the primary tumor with appropriate margins and elective nodal regions, including mesorectal, presacral, internal and external iliac, obturator, and inguinal lymph nodes, according to disease extent. Although the study spans a long period (2011–2024), the overall treatment strategy remained consistent, with IMRT-based chemoradiotherapy as the standard approach.

Over time, refinements mainly concerned improvements in imaging integration, image guidance, and treatment delivery, rather than substantial changes in target definition or dose prescription principles.

Dose–volume constraints for organs at risk followed NCCN guidelines and the RTOG 0529 protocol (5).

### 2.4 Boost strategy

Approximately four weeks after CRT, patients underwent clinical and radiological reassessment (MRI). Boost indication depended on initial stage and response, and was delivered either by interventional radiotherapy (IRT/brachytherapy) or external beam boost (ERT) (10, 11). The choice between interventional radiotherapy (IRT, brachytherapy and external beam boost (ERT) was based on a multidisciplinary assessment, taking into account baseline tumor stage, response to treatment, and anatomical feasibility.

IRT was generally preferred when focal dose escalation to a limited residual target was feasible and technically appropriate. External beam boost was selected in cases where IRT was not feasible due to anatomical constraints, tumor extent, or clinical conditions.

This approach reflects a personalized, response-adapted treatment strategy.

### 2.5 Follow-up and toxicity

Patients were evaluated weekly during treatment for acute toxicity (CTCAE v4.0). Follow-up visits were scheduled every 3 months for the first 2 years, every 6 months up to 5 years, and annually thereafter, including clinical examination and toxicity assessment. After completion of treatment, patients were followed according to institutional practice with regular clinical and radiological assessments. Pelvic MRI was the preferred imaging modality for locoregional evaluation, particularly in the early post-treatment phase, while CT and/or PET/CT were performed when clinically indicated for systemic assessment or in case of suspected recurrence.

Imaging was not fully uniform across all patients, reflecting the real-world nature of the study, but was guided by clinical judgment and standardized institutional pathways.

Clinical complete response (cCR) was defined as the absence of clinically detectable disease at physical examination, including digital rectal examination and inspection of the treated area.

Suspicion of recurrence was based on clinical findings, including new or worsening symptoms or abnormal physical examination, and/or radiological abnormalities detected during follow-up imaging. When clinically indicated, histological confirmation was obtained, particularly in cases considered for salvage treatment. This approach is consistent with previously published institutional experience and current clinical practice in anal cancer.

Late toxicities were evaluated according to CTCAE v4.0 score.

### 2.6 QoL assessment

Quality of life was assessed using patient-reported outcome measures (PROMs), including the Italian versions of EORTC QLQ-C30 and the anal cancer–specific module EORTC QLQ-ANL27 with permission from the EORTC QoL Group. The QLQ-C30 explores functional, symptom, and global QoL domains, while the QLQ-ANL27 focuses on bowel, pain, stoma, and sexual symptoms. Items are scored on 4-point.

Likert scales (1–4), except for the global QoL items (1–7) (12). Reference values for the Italian population were derived from Pilz et al. (13), which provide sex-, age-, and health-condition–adjusted reference values derived from a representative cohort of 1,036 individuals. These norms allowed benchmarking of survivors’ HRQoL against the expected values for the Italian population. Questionnaires were self-administered annually for up to five years after treatment completion. For patients who completed more than one QoL assessment during follow-up, only the most recent questionnaire in temporal order was included in the cross-sectional analysis, as it was considered representative of the long-term functional status.

For newly diagnosed patients, questionnaires were administered at baseline and during follow-up according to the study schedule. For patients already in follow-up at the time of study activation, PROMs were collected during follow-up visits to assess long-term survivorship.

### 2.7 Oncologic outcomes

Clinical complete remission (cCR) was defined as the absence of residual disease in the irradiated volume; late response was defined as occurring ≥26 weeks after CRT. Localand regional failures included both non-responders and the relapses after cCR. The recurrences were categorized as local (anal canal, anal margin, mesorectum) or regional (inguinal, iliac, obturator, perirectal, presacral nodes).

Colostomy-free survival (CFS) was calculated from the date of diagnosis to the positioning of permanent stoma or last follow-up and censored at reversal when applicable. Overall survival (OS) and disease-free survival (DFS) were defined from diagnosis to death and to loco-regional or distant recurrence, respectively.

### 2.8 Endpoints

The primary endpoint of the study was quality of life as assessed by PROMs.

Secondary endpoints included identification of clinical factors associated with QoL, assessment of OS,CFS, DFS, loco-regional control, and acute and late toxicity, as well as longitudinal analysis of QoL in long-term survivors (≥1 year). Associations between QoL outcomes and patient-, disease-, and treatment-related variables were also explored. The association between patient-, tumor-, and treatment-related variables and quality of life was.

### 2.9 Statistical analysis

Descriptive statistics were used to summarize patient characteristics and QoL scores. Statistical analyses were performed using IBM SPSS Statistics version 28.0 (IBM Corp., Armonk, NY, USA).

Data were summarized as absolute counts and percentages for categorical items while quantitative variables were reported as means and standard deviations or median and interquartile ranges (IQRs) depending on the distribution of the variables. Normality was assessed using the Kolmogorov-Smirnov test. According to the scoring manual, EORTC-C30 scales were linearly transformed to a 0–100 scale, with higher values indicating better functioning or greater symptom burden, depending on thedomain. Associations between EORTC-C30 scores and patient (sex, age ≤70 vs. >70, HIV status), disease(T, N stage, stoma), and treatment variables (dose, boost type, follow-up duration, toxicity) were analyzed using t-tests or ANOVA. Survival curves were estimated using Kaplan–Meier method. Two-sided p-values <0.05 were considered statistically significant.

Sample size was calculated according to the following steps assuming a level of significance of 5%, a total sample of 90 patients followed for at least 1 year provides 80% power to detect differences in EORTC-C30 between subgroups corresponding to an effect size of approximately 0.6. Assuming a standard deviation of 20.0 (derived from the reference population) this effect size corresponds to a difference of approximately 12 points, which represents a clinically meaningful change for patients. Due to the ambispective design and the availability of repeated PROM measurements only for a subset of patients, the primary QoL analysis was performed using a cross-sectional approach based on the most recent available questionnaire for each patient, in order to maximize data completeness and comparability.

Longitudinal trends in QoL were described when repeated measurements were available; however, these analyses were considered exploratory due to incomplete data across all time points.

## 3 Results

### 3.1 General scenario

A total of 90 patients were included. Patient, tumor and treatment characteristics are summarized in **Table 1**. The age at diagnosis ranged from 34 to 83 years (median, 62 years). There were 74 women (82.2%) and 16 men (17.8%); 6 patients (6.7%) were HIV-positive, and 25 (27.8%) were active smokers. Thirty-three patients were HPV-positive.

**Table 1:** Patients, tumor and treatment characteristics.

Variable	Value
Total n of patients	90 (100%)
SEX Male	16 (17.8)
SEX Female	74 (82.2)
AGE AT DIAGNOSIS in years (median, IQR)	62 (54-68)
BMI (median, IQR)	23.6 (21.6-27.0)
GRADING	
Gx	53 (58.9)
G1	7 (7.8)
G2	11 (12.2)
G3	19 (21.1)
HPV	
Negative	10 (11.1)
Positive	33 (36.6)
missing	47 (52.2)
T STAGE	
Tx	7 (7.8)
T1	4 (4.4)
T2	36 (40.0)
T3	17 (18.9)
T4	26 (28.9)
N STAGE	
N0	33 (36.7)
N1	41 (45.6)
N2	2 (2.2)
N3	14 (15.6)
M STAGE	
M0	86 (95.6)
M1	4 (4.4)
STAGE	
0	1 (1.1)
1	3 (3.3)
2	22 (24.4)
3	60 (66.7)
4	4 (4.4)
HIV	
Negative	84 (93.3)
Positive	6 (6.7)
SMOKING HABITS	
Never	65 (72.2)
Current	12 (13.3)
Former	13 (14.4)
CHEMOTHERAPY	
No	2 (2.2)
FUMIR (5fu-mmc)	79 (87.8)
PLAFUR (cddp-5fu)	4 (4.4)
Other	5 (5.5)
NUMBER OF CT CYCLE (median, range)	2 (1-6)
EBRT TOTAL DOSE (median, IQR)	54.0 (50.0-55.0)
BOOST	
No	27 (30.0)
IBRT	50 (55.6)
EBRT	13 (14.4)

RCT, radiochemotherapy; IBRT, interstitial brachytherapy; EBRT, external beam radiotherapy; MMC, mitomycin, 5-FU 5-fluorouracil.

All patients, except two, underwent concurrent chemoradiotherapy (CRT). The remaining two received radiotherapy alone due to significant comorbidities that contraindicated systemic treatment.

The EBRT dose ranged from 41.4 to 60 Gy (median, 51.7 Gy). Thirty-five (%) patients experienced treatment interruptions (mean 4.8 days), of whom 22 had interruptions longer than three days. The most common causes of interruptions longer than three days were hematologic and cutaneous G1-G2 toxicities.

A dose boost was delivered in 63 (%) patients: specifically, 50 patients received IRT boost, 33 received a single fraction of 4 Gy and 17 received two fractions of 4 Gy one week apart, while the remaining 13 patients were boosted with external beam radiotherapy.

The median follow-up time was 51 months (range, 14–118 months). Gastrointestinal (GI) toxicity represented the most frequent late adverse event, with grade 1 in 28 patients (31.1%), grade 2 in 18 (20.0%), and grade 3 in 6 (6.7%). The most reported GI symptoms included mild to moderate anal pain, tenesmus, stool urgency, increased bowel frequency, and occasional episodes of fecal incontinence.

Genitourinary (GU) toxicity was generally mild, with grade 1 in 16 patients (17.8%) and grade 2 in 5 (5.6%); no grade ≥3 events were observed.

Late skin toxicity was infrequent, recorded as grade 1 in 5 patients (5.6%), grade 2 in 1 (1.1%), and grade 3 in 1 (1.1%).

A permanent stoma, due either to disease recurrence or to a non-functioning sphincter, was present in 14 patients (15.5%).

### 3.2 Survival analysis

Three- and five-year overall survival were both 97.5%. Disease-free survival was 88.2% and 84.5% at three and five years, respectively, while locoregional relapse-free survival was 92.8% and 89.0%.

Colostomy-free survival showed identical rates at three and five years (97.3%) (**Supplementary Files**).

### 3.3 Quality of life PROMs

Patients were included in the cross-sectional QoL analysis using the most recent available questionnaire.

Among these 90, 41 patients had repeated PROM assessments over time; however, longitudinal changes were not formally analyzed and are considered descriptive.

To evaluate long-term functional outcomes, all patients who had been in follow-up for at least one year completed the EORTC QLQ-C30 v3 and EORTC QLQ-ANL27 questionnaires during follow-up. In both EORTC QLQ-C30 v3 and EORTC QLQ-ANL27 questionnaires, functional and symptoms scale were listed as illustrated in **Tables 2**–**4**. Overall, global QoL appeared generally preserved in the study population. However, domain-specific impairments were observed, highlighting the heterogeneous impact of treatment on long-term survivorship.

**Table 2:** EORTC QLQ-C30 v3 functional scales.

Functional scale	Mean score	Standard Deviation (SD)
Overall Quality of Life (QoL)	74.16	16.62
Physical Functioning (PF)	84.9	17.27
Role Functioning (RF)	85.5	21.95
Emotional Functioning (EF)	78.2	20.88
Cognitive Functioning (CF)	85.3	22.18
Social Functioning (SF)	85.0	23.31

**Table 3:** EORTC QLQ-C30 v3 symptom scales and single items.

Symptom scale / Item	Mean score	Standard deviation (SD)
Fatigue (FA)	24.4	23.62
Pain (PA)	14.0	21.33
Nausea/Vomiting (NV)	1.6	5.61
Dyspnea (DY)	11.1	23.96
Insomnia (SL)	20.7	26.71
Appetite Loss (AP)	3.3	10.05
Constipation (CO)	13.7	21.72
Diarrhea (DI)	17.0	23.56
Financial Difficulties (FI)	13.3	27.27

**Table 4:** EORTC QLQ-ANL27 symptom and functional scales.

Scale / Item	Domain	Mean score	Standard deviation (SD)
Bowel Symptoms – Non-Stoma (BOW_NS)	Symptom	30.13	22.74
Bowel Symptoms – Stoma (BOW_S)	Symptom	11.31	14.83
Stoma Care (STO)	Symptom	11.91	15.99
Pain (PAIN)	Symptom	12.98	15.81
Urinary Frequency (UF)	Symptom	18.96	26.16
Swelling in Legs/Ankles (SWE)	Symptom	17.25	27.30
Need to Be Close to a Toilet (WC)	Symptom	19.48	29.64
Cleaning Oneself More Often (CL)	Symptom	23.49	29.10
Planning Activities (PL)	Symptom	16.28	29.70
Sexual Life and Interest – Men (SEX_M)	Function	53.70	23.42
Sexual Life and Interest – Women (SEX_F)	Function	55.53	28.39

Bowel-related symptoms showed clinically relevant differences across patient subgroups.

In particular, younger patients (<70 years) reported higher symptom burden compared with older patients (mean score 32.2 vs 21.0), corresponding to a difference of approximately 11 points, consistent with a clinically meaningful variation, although not reaching statistical significance (p=0.09) (see **Supplementary Table 5**). Sexual function emerged as one of the most affected domains. Female patients aged <70 years reported significantly worse outcomes compared with older patients (mean score 51.2 vs 72.2), with a difference exceeding 20 points, indicating a clinically meaningful impairment (p=0.02) (also see **Supplementary Table 5**).

In contrast, differences in male sexual function were not statistically significant.

Other symptom domains, including pain and urinary function, showed only modest differences between groups, generally below thresholds considered clinically meaningful.

### 3.4 Comparison with general population

**Figure 1:**
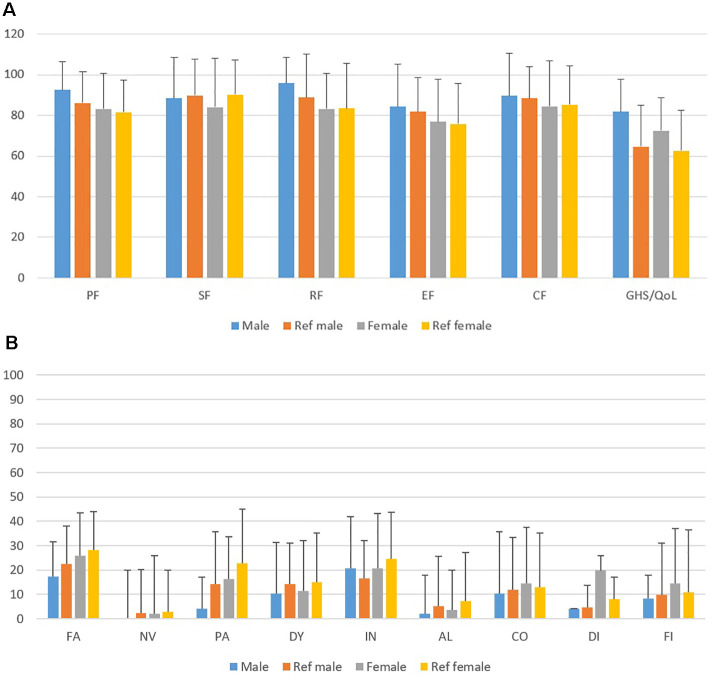
**(A, B)** EORTC QLQ-C30 functional and symptoms scales in the study cohort compared with Italian sex-matched normative values. PF, physical function; RF, role function; CF, cognitive function; EF, emotional function; QoL, global quality of life; FA, fatigue; NV, nausea/vomiting; PA, pain; DY, dyspnea; IN, insomnia; AL, loss of appetite; CO, constipation; DI, diarrhea; FI, financial problems.

**Table 5** reports the mean (± SD) QLQ-C30 domain scores in the study cohort, stratified by sex, compared with the corresponding normative values from the Italian general population aged ≥70 years, together with the respective *p*-values (13). Global Health Status/QoL was significantly better in both men and women (*p* = 0.002 and *p* = 0.001). Symptom scales were overall comparable to normative values, except for diarrhea in female patients (*p* = 0.0008), likely reflecting mild persistent pelvic toxicity (**Figure 1**).

**Table 5:** Functioning and symptoms scale of the EORTC QLQ C30 patients versus Italian reference population.

Scale/item	Male	Male pop (n=71)	P value	Female	Female pop (n=77)	P value
PF	92.5 (14.2)	86.1 (15.6)	0.13	83.3 (17.5)	81.7 (15.8)	0.56
SF	88.5 (19.9)	89.9 (18.0)	0.78	84.2 (24.0)	90.3 (17.1)	0.07
RF	95.8 (12.9)	88.9 (21.4)	0.22	83.3 (17.5)	83.5 (22.3)	0.95
EF	84.3 (20.8)	81.8 (16.9)	0.61	76.9 (20.8)	75.8 (20.1)	0.74
CF	89.6 (21.0)	88.4 (15.6)	0.79	84.5 (22.5)	85.3 (19.0)	0.81
GHS/QoL	81.8 (15.9)	64.8 (20.4)	0.002	72.5 (16.4)	62.7 (20.0)	0.001
FA	17.3 (25.3)	22.4 (21.5)	0.41	26.0 (23.1)	28.2 (22.2)	0.55
NV	0 (0)	2.3 (9.2)	--	2.0 (6.1)	2.8 (9.2)	0.53
PA	4.2 (9.6)	14.2 (21.2)	0.07	16.2 (22.6)	22.8 (25.5)	0.09
DY	10.4 (20.1)	14.2 (23.3)	0.55	11.3 (24.8)	15.0 (21.4)	0.33
IN	20.8 (26.9)	16.5 (23.0)	0.51	20.7 (26.9)	24.7 (26.7)	0.36
AL	2.1 (8.3)	5.3 (16.2)	0.45	3.6 (10.4)	7.3 (15.4)	0.09
CO	10.4 (16.0)	11.9 (21.9)	0.80	14.4 (22.8)	13.0 (20.6)	0.69
DI	4.2 (11.4)	4.6 (13.4)	0.91	19.8 (24.6)	8.0 (17.2)	0.0008
FI	8.3 (25.8)	9.9 (21.9)	0.80	14.4 (27.6)	11.0 (22.8)	0.41

PF, physical function; RF, role function; CF, cognitive function; EF, emotional function; QoL, global Quality of life; FA, fatigue; NV, nausea/vomiting; PA, pain; DY, dyspnea; IN, insomnia; AL, loss of appetite; CO, constipation; DI, diarrhea; FI, financial problems.

### 3.5 Associations between QoL outcomes and clinical variables

The association between patient-, tumor-, and treatment-related variables and quality of life was assessed using the EORTC QLQ-C30 and QLQ-ANL27 questionnaires (see **Supplementary Appendix Tables**). In the QLQ-C30 analysis, among patient-related variables, younger age (<70 years) correlated with better physical functioning (*p* = 0.049), while sex differences were evident: men reported higher role functioning (*p* = 0.005) and global QoL (*p* = 0.043), whereas women showed higher pain (*p* = 0.001) and diarrhea scores (*p* < 0.001). When correlated with late gastrointestinal (GI) toxicity, several domains showed significant impairment. Patients with late GI symptoms had lower physical, role, and social functioning (*p* = 0.001, <0.001, and 0.032, respectively) and higher fatigue, pain, dyspnea, and diarrhea scores (*p* = 0.005, 0.001, 0.03, and <0.001, respectively). In contrast, global QoL was significantly higher in patients without late GI toxicity (*p* = 0.001) Late genitourinary (GU) toxicity correlated only with higher pain (*p* = 0.03).

In the disease-specific QLQ-ANL27, the influence of demographic and toxicity-related factors was more pronounced. Female patients reported significantly worse symptom-related outcomes, with higher scores for bowel symptoms (BOW_NS, p = 0.004), pain (p < 0.001), need to be close to a toilet (WC, p = 0.013), and cleaning oneself more often (CL, p < 0.001), while planning activities (PL) showed a non-significant trend (*p* = 0.24). Conversely, men had better role functioning and global QoL.

Age under 70 years was associated with poorer sexual functioning among women (*p* = 0.02) (**Table 6**). The presence of late GI toxicity was the most impactful variable, correlating with significantly worse scores in BOW_NS (p < 0.001), pain (p = 0.005), WC (p < 0.001), CL (p < 0.001), and PL (p = 0.01), as well as reduced female sexual functioning (p = 0.02).

**Table 6:** EORTC QLQ-ANL27 scores by sex (mean ± SD).

Scale	Men (M) N	Mean	SD	Women (F) N	Mean	SD	p-value
BOW_NS	15	19.5	12.0	60	32.8	24.1	0.004
BOW_S	1	16.7	–	13	10.9	15.4	–
PAIN	16	3.7	5.1	73	15.0	16.6	<0.001
STO	1	0.0	–	13	12.8	16.3	–
UF	16	20.9	29.6	72	18.5	25.6	0.75
SWE	16	12.5	20.6	71	18.3	28.6	0.44
WC	16	8.3	14.9	73	21.9	31.5	0.013
CL	16	8.3	14.9	72	26.9	30.5	<0.001
PL	16	8.3	25.8	70	18.1	30.4	0.24
SEX	15	53.7	23.4	–	–	–	–
SEX	–	–	–	58	55.5	28.4	–

BOW_NS, bowel symptoms non-stoma; BOW_S, bowel symptoms stoma; STO, stoma care; PAIN, pain; UF, urinary frequency; SWE, swelling in legs/ankles; WC, need to be close to toilet; CL, cleaning oneself more often; PL, planning activities; SEX_M, sex life and interest (men); SEX_F, sex life and interest (women); SD, standard deviation.

Although a formal minimally clinically important difference (MCID) analysis was not performed, the magnitude of differences observed in selected domains—particularly bowel symptoms and sexual function—was consistent with clinically meaningful changes based on established EORTC interpretation guidelines (**Table 7**).

**Table 7:** EORTC QLQ-ANL27 scores by late gastrointestinal toxicity (mean ± SD).

Scale	GI late no (N)	Mean	SD	GI late yes (N)	Mean	SD	p-value
BOW_NS	29	16.7	14.3	46	38.6	23.1	<0.001
BOW_S	9	6.5	8.1	5	20.0	20.9	0.23
PAIN	38	7.6	15.7	51	17.0	14.8	0.005
STO	9	9.9	18.0	5	15.6	12.7	0.55
UF	38	13.2	22.7	50	23.4	27.9	0.06
SWE	38	14.9	27.6	49	19.1	27.2	0.48
WC	38	7.9	16.3	51	28.1	34.2	<0.001
CL	38	8.8	18.5	50	34.7	30.8	<0.001
PL	36	7.4	19.7	50	22.7	34.0	0.010
SEX_M	8	54.5	26.9	7	52.8	20.8	0.89
SEX_F	26	65.1	25.2	32	47.7	28.8	0.02

BOW_NS, bowel symptoms non-stoma; BOW_S, bowel symptoms stoma; STO, stoma care; PAIN, pain; UF, urinary frequency; SWE, swelling in legs/ankles; WC, need to be close to toilet; CL, cleaning oneself more often; PL, planning activities; SEX_M, sex life and interest (men); SEX_F, sex life and interest (women); SD, standard deviation.

## 4 Discussion

### 4.1 Oncologic outcomes and treatment tolerance in the IMRT era

In this ambispective cohort, IMRT-based chemoradiotherapy (CRT), with selective use of image-guided interventional radiotherapy (IRT) boost, was associated with promising long-term oncologic outcomes. At a median follow-up of 51 months, 3- and 5-year overall survival were both 97.5%, disease-free survival was 88.2% and 84.5%, respectively, and colostomy-free survival reached 97.3%. Severe late toxicity was uncommon, with grade ≥3 gastrointestinal events observed in 6.7% of patients.

PROACT represents one of the first Italian experience reporting long-term oncologic outcomes in the era of IMRT and image-guided IRT with results consistent with major IMRT based trials, including RTOG 0529, and institutional series incorporating image-guided boost strategies (5, 6, 9, 10). Compared with historical pre-IMRT cohorts, in which 5-year overall survival ranged between 65% and 70% and morbidity was substantial, outcomes observed in PROACT appear favorable and consistent with those reported in modern IMRT-based series (25–27).

The high survival rates observed in PROACT may reflect the systematic adoption of IMRT, optimized target delineation, and dose modulation, together with selective image-guided dose escalation, potentially allowing effective tumor control while limiting high-grade late toxicity (19).

Oncologic efficacy and sphincter preservation were observed without an apparent increase in severe adverse events, in line with the favorable therapeutic ratio of contemporary conformal approaches.

These excellent oncologic outcomes and low rates of severe late toxicity provide a robust foundation for the evaluation of long-term survivorship and patient-reported outcomes beyond clinician-reported endpoints.

### 4.2 Patient-reported outcomes and survivorship burden

Despite favorable clinician-reported toxicity profiles, patient-reported outcomes (PROMs) revealed a broader and more nuanced burden of long-term functional sequelae, highlighting a clear discordance between CTCAE-based assessments and patients’ lived experience. This finding supports previous evidence suggesting that conventional toxicity scoring may underestimate the real life impact of treatment on daily functioning and well-being (7). The combined use of the EORTC QLQC30 and the disease-specific QLQ-ANL27 enabled a comprehensive assessment of survivorship, capturing clinically relevant late effects that may not be adequately reflected by generic quality-of-life measures alone. In particular, disease-specific domains related to bowel function, toileting dependence, and sexual health emerged as key drivers of long-term symptom burden (12, 13, 17). Randomized evidence from the PLATO- ACT4 trial further supports the value of PROMs in detecting subtle but clinically meaningful differences in late toxicity and functional recovery (18).

In our cohort, approximately one-third of long-term survivors reported persistent bowel dysfunction and toileting dependence, while fatigue and sexual difficulties remained prevalent beyond a median follow-up of more than four years. These results are consistent with previous prospective and qualitative studies showing that survivorship in anal canal cancer is frequently characterized by chronic functional symptoms despite durable disease control (2–4, 8, 14–16).

Importantly, interindividual variability in symptom perception and functional adaptation was observed, reflecting a dimension of resilience that allows acceptable global health status scores to coexist with persistent, domain-specific impairments. This dissociation helps explain why overall quality-of-life measures may remain preserved despite clinically meaningful functional limitations (7).

Together, these findings underscore the critical role of PROMs in complementing clinician reported toxicity and provide the rationale for a patient-centered evaluation of long-term survivorship outcomes.

### 4.3 Quality of life in context and key factors associated with impairment

When benchmarked against age- and sex-matched Italian normative data, PROACT survivors reported preserved, and in some domains even higher, Global Health Status and overall quality-of-life scores, while most functional scales were comparable to those observed in the general population (p < 0.05) (13) (see **Table 5**).

In line with contemporary IMRT-based literature, this favorable long-term profile suggests that modern multidisciplinary management, including IMRT and image-guided strategies, is associated with recovery of global functioning after curative treatment (6, 18).

However, quality-of-life recovery was not uniform across patient subgroups. In univariate analyses female sex, younger age (<70 years), and the presence of late gastrointestinal toxicity emerged as relevant factors associated with impaired patient-reported outcomes across both the EORTC QLQ-C30 and QLQ-ANL27. Late gastrointestinal toxicity was strongly associated with reduced physical, role, and social functioning, as well as increased symptom burden, confirming previous reports that symptom persistence is a key mechanism of long-term quality-of-life deterioration (14, 15).

Women—particularly younger survivors—reported worse bowel symptoms, greater toileting dependence, higher pain scores, and poorer sexual functioning, indicating a disproportionate impact on psychosocial well-being and body image, thereby identifying a clinically vulnerable subgroup rather than implying a causal relationship. These findings are in line with prior IMR based survivorship studies and reinforce the concept that anatomical preservation and disease control do not necessarily translate into full functional recovery in vulnerable subgroups (7, 14, 16, 20).

Compared with the recent experience of Jirkovská et al., which reported long-term quality-of- life outcomes in a similarly treated IMRT-based cohort with comparable treatment intent and PROMs (20), our PROACT cohort achieved slightly higher global and functional scores (GHS 74.2; FS 89.8 vs. GHS 68.4; FS 86.2). These differences may reflect, at least in part, the younger age of our population and the use of contemporary IMRT ± image- guided IRT boost, which allows greater conformality and sphincter sparing. Both experiences confirm excellent long-term functional recovery and suggest that image-guided dose escalation may be a feasible strategy without apparent detrimental impact on quality of life. (Interestingly, while Jirkovská et al. identified age >70 as the main determinant of poorer physical functioning and global health, in our PROACT series older patients maintained overall good QoL, with global and functional scores (GHS 71.5; PF2 86.1) comparable to those of younger counterparts. This observation suggests that the systematic use of IMRT and careful dose modulation of the anal sphincter complex may be associated with preservation of quality of life across age groups. These findings reinforce the concept that optimized image- guided radiotherapy may contribute to preserving functional integrity even in elderly patients, effectively broadening the therapeutic window of curative-intent treatment.

Taken together, these studies and the present findings consistently emphasize the need to interpret oncologic success within the lived experience of survivors.

### 4.4 Clinical implications and limitations

From a clinical perspective, these findings support a shift from an exclusive focus on tumor control and organ preservation toward a survivorship-oriented care model that integrates long term functional, emotional, and social outcomes. The routine incorporation of patient-reported outcome measures (PROMs), particularly disease-specific instruments such as the EORTC QLQ-ANL27, into follow-up enables a more comprehensive assessment of late effects and facilitates the early identification of vulnerable subgroups, including younger women and patients experiencing late gastrointestinal toxicity (7, 18, 21). Within this framework, targeted supportive and rehabilitative interventions, including bowel, sexual health, and psychosocial support, should be considered integral components of post-treatment care. Furthermore, the selective use of image-guided interventional radiotherapy (IRT) boost may represent a valuable strategy without an apparent decrease in severe morbidity in this cohort, supporting its potential role as part of a tailored risk-adapted treatment approach. In our experience, focal dose escalation using IRT allowed treatment intensification in selected patients with minimal additional morbidity, supporting its role as part of a tailored, risk-adapted treatment approach (11, 20, 22, 23).

This study has several limitations. The absence of baseline quality-of-life assessments precludes a full evaluation of treatment-related changes over time, while the cross-sectional nature of the most recent PROMs assessment limits longitudinal interpretation of survivorship trajectories. In addition, the lack of detailed dosimetric–functional correlations restricts a more granular understanding of the relationship between radiation dose distribution and long-term functional outcomes. Future studies should therefore incorporate prospective baseline and longitudinal PROM collection, integrate dosimetric and functional analyses, and develop structured, multidisciplinary survivorship pathways. Such approaches will be essential to further refine treatment strategies and optimize long- term quality of life in patients treated with curative intent for anal canal cancer.

These findings should be interpreted with caution given the observational design, the limited sample size, and the predominantly univariate analyses, and should therefore be considered hypothesis-generating.

## 5 Conclusions

In this study, quality of life in patients treated with curative intent for anal canal cancer was evaluated using integrated patient-reported outcome measures. Compared with age- and sex-matched Italian normative data, PROACT survivors reported higher global quality-of-life and functional scores, suggesting substantial recovery and long-term adaptation after IMRT-based chemoradiotherapy.

However, detailed PROM assessment using the EORTC QLQ-ANL27 revealed persistent bowel and sexual symptoms, particularly among women under 70 years and patients with late gastrointestinal toxicity—subgroups that may benefit from focused supportive and rehabilitative interventions.

These findings underscore that long-term treatment success extends beyond disease control and includes sustained functionality and patient-reported well-being. Moreover, evidence from other disease settings suggests that relevant quality-of-life issues may persist across the anal cancer continuum, reinforcing the need for stage-inclusive, patient-centered PROM integration in clinical practice (24).

## Data Availability

The original contributions presented in the study are included in the article/**Supplementary Material**. Further inquiries can be directed to the corresponding author.
