# Supplementary material for: “Surviving is not enough”: shifting the focus from treatment success to quality of life in anal cancer survivors. Patient- reported outcomes and the evolving landscape of survivorship care
Source: Front Oncol. 2026 May 19;16:1761654. doi: 10.3389/fonc.2026.1761654 (PMC13225979; doi:10.3389/fonc.2026.1761654)
Supplement: Supplementary file 1 [file DataSheet1.docx]

Supplementary table 4. Associations between age and EORTC QLQ C30

PF physical function, RF role function, EF emotional function, CF cognitive function, SF social function, QOL quality of life, FA fatigue, NV nausea/vomiting, PA pain, DY dyspnea, SL insomnia, AP loss of appetite, CO constipation, DI diarrhea, FI financial difficulties)

|  | età | N | Mean | Std. Deviation |  |
| --- | --- | --- | --- | --- | --- |
| BOW_NS | <70 | 61 | 32.235 | 23.3991 | 0.09 |
|  | >=70 | 14 | 20.952 | 17.5133 |  |
| BOW_S | <70 | 10 | 14.167 | 16.2209 | 0.27 |
|  | >=70 | 4 | 4.167 | 8.3333 |  |
| PAIN | <70 | 71 | 13.207 | 16.3816 | 0.79 |
|  | >=70 | 18 | 12.099 | 13.7457 |  |
| STO | <70 | 10 | 8.889 | 11.4755 | 0.28 |
|  | >=70 | 4 | 19.444 | 24.6373 |  |
| UF | <70 | 70 | 20.019 | 26.8571 | 0.45 |
|  | >=70 | 18 | 14.815 | 23.4931 |  |
| SWE | <70 | 69 | 17.406 | 27.1726 | 0.92 |
|  | >=70 | 18 | 16.667 | 28.5831 |  |
| WC | <70 | 71 | 20.188 | 31.1060 | 0.65 |
|  | >=70 | 18 | 16.667 | 23.5702 |  |
| CL | <70 | 70 | 25.238 | 29.7254 | 0.27 |
|  | >=70 | 18 | 16.667 | 26.1968 |  |
| PL | <70 | 70 | 16.667 | 30.4290 | 0.80 |
|  | >=70 | 16 | 14.583 | 27.1314 |  |
| SEXM | <70 | 13 | 51.496 | 24.1793 | 0.37 |
|  | >=70 | 2 | 68.056 | 13.7493 |  |
| SEXF | <70 | 46 | 51.171 | 28.3120 | 0.02 |
|  | >=70 | 12 | 72.222 | 22.7217 |  |

Supplementary table 5. Association between age and EORTC ANL 27

bowel symptoms non stoma BOW_NS, bowel symptoms
[_S), stoma care (STO), pain (PAIN), urinary frequency (UF), swelling in legs/ankles
(SWE), need to be close to a toilet (C), cleaning one self more oftea (CL), plaming activities (PL), sex life and interest-men (SEX M) and women (SEX F)

Supplementary table 6 Associations between late GI and EORTC QLQ C30

PF physical function, RF role function, EF emotional function, CF cognitive function, SF social function, QOL quality of life, FA fatigue, NV nausea/vomiting, PA pain, DY dyspnea, SL insomnia, AP loss of appetite, CO constipation, DI diarrhea, FI financial difficulties)


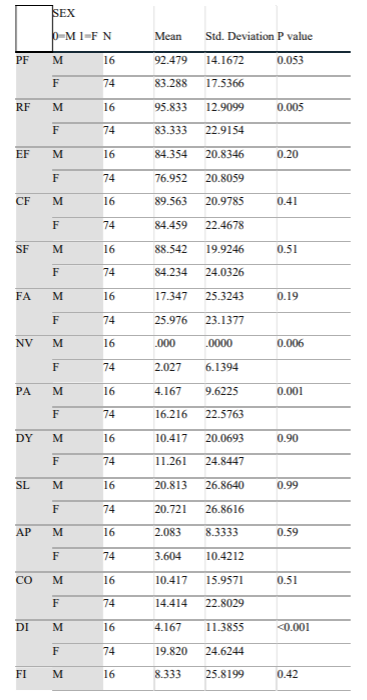


Supplementary table 7 Associations between Sex and EORTC QLQ C30

PF physical function, RF role function, EF emotional function, CF cognitive function, SF social function, QOL quality of life, FA fatigue, NV nausea/vomiting, PA pain, DY dyspnea, SL insomnia, AP loss of appetite, CO constipation, DI diarrhea, FI financial difficulties)


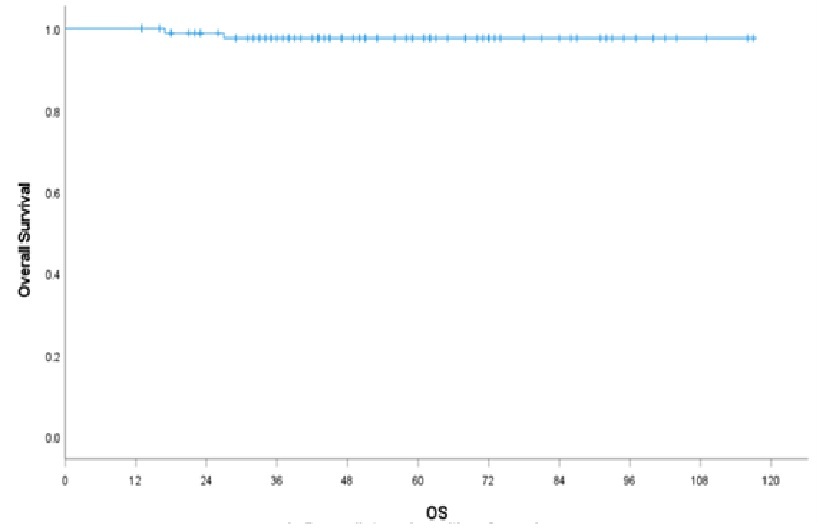


Supplementary Figure 1a. Overall survival


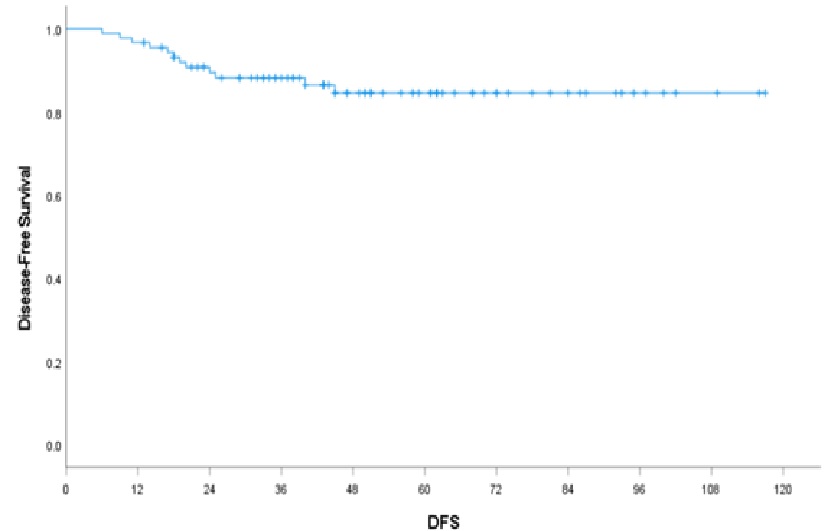


Supplementary Figure 1b. Disease Free Survival


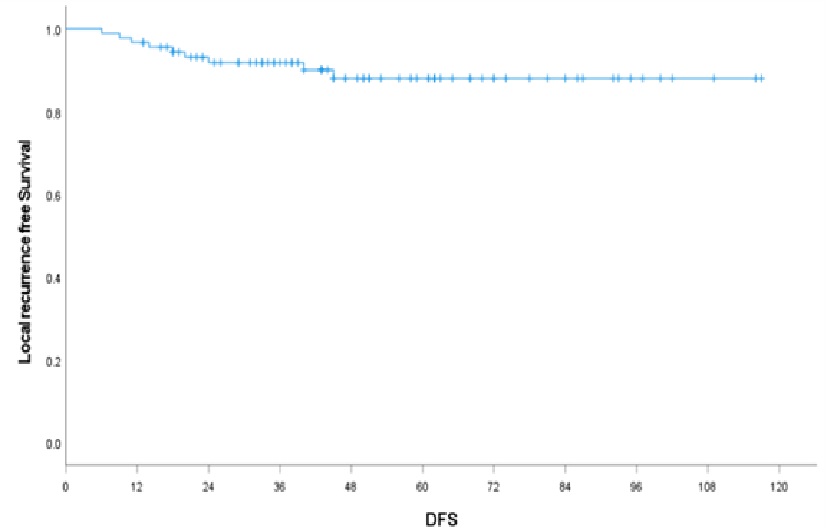


Supplementary Figure 1c. Local Recurrence Free Survival


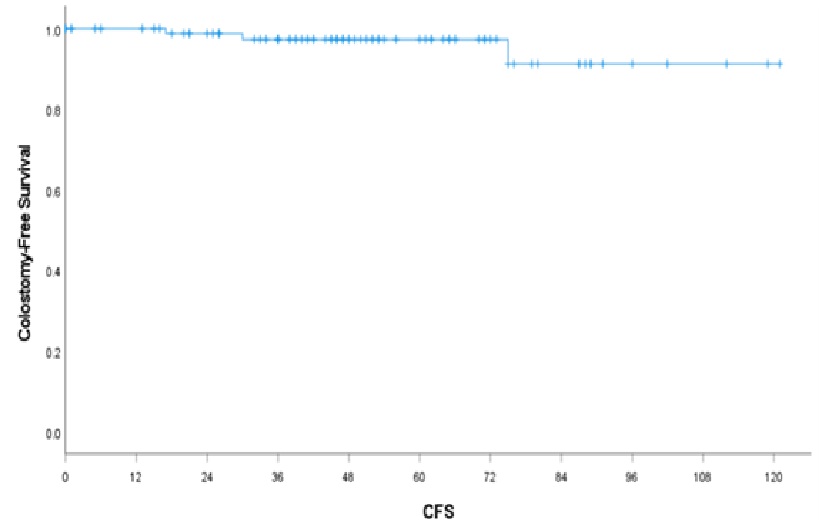


Supplementary Figure 1d. Colostomy Free Survival
